# Survival Benefit of Adjuvant Radiation Therapy for Gastric Cancer following Gastrectomy and Extended Lymphadenectomy

**DOI:** 10.1155/2012/307670

**Published:** 2012-06-21

**Authors:** R. A. Snyder, E. T. Castaldo, C. E. Bailey, S. E. Phillips, A. B. Chakravarthy, N. B. Merchant

**Affiliations:** ^1^Department of Surgery, Vanderbilt University Medical Center, Nashville, TN 37232, USA; ^2^Department of Biostatistics, Vanderbilt University Medical Center, Nashville, TN 37232, USA; ^3^Department of Radiation Oncology, Vanderbilt University Medical Center, Nashville, TN 37232, USA; ^4^Vanderbilt Ingram Cancer Center, Nashville, TN 37232-6860, USA; ^5^Division of Surgical Oncology, Vanderbilt University Medical Center, 597 Preston Research Building, Nashville, TN 37232, USA

## Abstract

*Purpose*. Although randomized trials suggest a survival benefit of adjuvant chemotherapy and radiation therapy (XRT) for gastric adenocarcinoma, its use in patients who undergo an extended lymphadenectomy is less clear. The purpose of this study was to determine if a survival benefit exists in gastric cancer patients who receive adjuvant XRT following resection with extended lymphadenectomy. *Methods*. The SEER registry was queried for records of patients with resected gastric adenocarcinoma from 1988 to 2007. Multivariable Cox regression models were used to assess independent prognostic factors affecting overall survival (OS) and disease-specific survival (DSS). *Results*. Of 15,060 patients identified, 3,208 (21%) received adjuvant XRT. Adjuvant XRT was independently associated with improved OS (HR 0.67, CI 0.64–0.71) and DSS (HR 0.69, CI 0.65–0.73) in stages IB through IV (M0). This OS and DSS benefit persisted regardless of the extent of lymphadenectomy. Furthermore, lymphadenectomy with >25 LN resected was associated with improved OS and DSS compared with <15 LN or 15–25 LN. *Conclusion*. This population-based study shows a survival benefit of adjuvant XRT following gastrectomy that persists in patients who have an extended lymphadenectomy. Furthermore, removal of >25 LNs results in improved OS and DSS compared with patients who have fewer LNs resected.

## 1. Introduction

Gastric cancer is the fourth most common cancer and second leading cause of cancer-related deaths worldwide [1]. It has been estimated that there were 21,000 new cases of gastric cancer and 10,570 deaths from gastric cancer in the United States in 2010 [2]. Most patients in the U.S. present with locally advanced disease in which the tumor penetrates the muscularis propria and/or involves the perigastric lymph nodes at the time of diagnosis [3]. Surgical resection remains the only curative option for gastric adenocarcinoma. However, locoregional and systemic recurrence rates remain high, and ten-year overall survival (OS) rates after resection with curative intent range from 3–42% for advanced disease [4–6]. Given the high rates of recurrence after resection, the additional use of adjuvant chemotherapy and radiation therapy (XRT) has been investigated.

In 2001, the US Intergroup study (INT-0116) demonstrated an improvement in OS for patients with stage IB through IV (M0) gastric cancer who underwent resection followed by adjuvant 5-FU-based chemotherapy and XRT compared with patients who underwent surgical resection alone [7]. Based on these results, this adjuvant therapy regimen became the standard of care for resectable gastric cancer in the U.S. A retrospective observational study using the Surveillance, Epidemiology, and End Results (SEER)—Medicare database reproduced these findings, demonstrating an improvement in OS for patients with gastric cancer who received adjuvant chemoradiation therapy [8].

In the INT-0116 trial, a D2 lymph node dissection (resection of regional lymphatics and perigastric lymph nodes (LNs), as well as LNs along the named vessels of the celiac axis) was recommended in all patients. However, only 10% of patients actually received this level of nodal clearance. In fact, 54% of patients underwent a D0 resection (gastrectomy with incomplete resection of the N1 nodes) [7]. This calls into question whether adjuvant chemoradiation therapy was associated with improved disease-free survival (DFS) and OS in this trial by decreasing locoregional recurrence in patients who underwent an inadequate lymph node dissection (LND). In a post hoc analysis of INT-0116, there was no significant evidence that chemoradiation failed to work in the D2 subgroup; however, with only 54 patients in this group, the authors acknowledge that the power of this analysis was very low [9].

The primary aim of this study, therefore, was to evaluate whether the survival benefit of adjuvant chemoradiation therapy persists in patients undergoing gastrectomy and extended lymphadenectomy by using a large, national database that could provide significant statistical power to detect a survival difference.

## 2. Materials and Methods

A data set consisting of patients with gastric adenocarcinoma was created through a query of the SEER database. SEER is an authoritative source for cancer incidence, survival, and prevalence encompassing approximately 28% of the United States population [10]. The SEER program collects demographic information (e.g., age, gender, and race) and clinical information (e.g., primary tumor site, tumor histology, tumor grade, stage, treatment, and survival) from 17 cancer registries across the United States. Stage information from the SEER database was converted to the American Joint Committee on Cancer 6th edition (AJCC) tumor node metastasis (TNM) criteria. 

All patient records in the SEER registry from 1988 to 2007 with surgically resected gastric adenocarcinoma were queried (*n* = 78,511). Patients in whom gastric adenocarcinoma was not the primary malignancy or patients who lacked a histologically confirmed diagnosis of gastric adenocarcinoma were excluded from analysis. Patients with gastric lymphoma, gastrointestinal stromal tumors, or other gastric malignancies were excluded. In addition, those patients who had incomplete clinicopathologic information, metastatic disease, or who had undergone preoperative or intraoperative XRT were excluded.

Statistical analysis was performed using a Cox regression model with gender, race (categorized as white, black, and other), age, number of LNs resected, AJCC stage of disease, and radiation status as covariates. The primary and secondary outcome measures were OS and DSS, respectively. Kaplan-Meier methods and the log-rank test were also used to compare survival between patients who did or did not receive adjuvant XRT according to stage and extent of LND. For purposes of statistical analysis, the number of LNs resected was subdivided into groups of <15 LNs, 15–25 LNs, and >25 LNs. Age was dichotomized to ≤60 years and >60 years of age. All *P* values were two-tailed tests, with alpha of 0.05.

The Vanderbilt Institutional Review Board (IRB) was contacted regarding this study; however, because the data from SEER is de-identified prior to release to our institution, no formal IRB approval was required. 

## 3. Results

A total of 15,060 patients met the inclusion criteria. Of these, 3,208 (21%) received adjuvant XRT after gastric resection and 11,852 (79%) underwent gastric resection alone. Patient characteristics are shown in Table 1. Several statistical differences between patient populations were identified. Patients of younger age (≤60), male gender, and higher stage tumors were more likely to receive adjuvant XRT (*P* < 0.001). Patients who had ≥15 LNs removed were also more likely to receive adjuvant XRT (*P* < 0.001).

Kaplan Meier survival analysis demonstrated a significant 5 year OS benefit of adjuvant XRT when compared with surgery alone for all patients with AJCC stage IB (*P* = 0.002) and higher (*P* < 0.001). Median OS among patients with stage IB, II, IIIA, and IIIB gastric cancer who received adjuvant XRT was 65, 34, 23, and 19 months, respectively, compared with 54, 21, 14, and 11 months, respectively, for patients who underwent surgery alone. These results are summarized in Table 2. Five year DSS was also improved for patients with stage II and higher who received adjuvant XRT (*P* < 0.001). Median DSS for patients who received adjuvant XRT with stages II, IIIA, and IIIB was 41, 24, and 20 months, respectively, compared with 26, 17, and 12 months for patients who had surgery alone.

**Figure 1:**
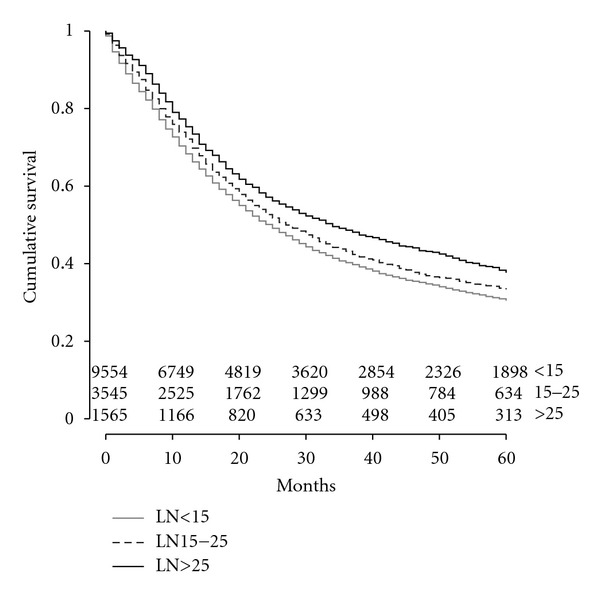
Kaplan Meier curve of overall survival by lymph node resection. Lymph node (LN).

**Figure 2:**
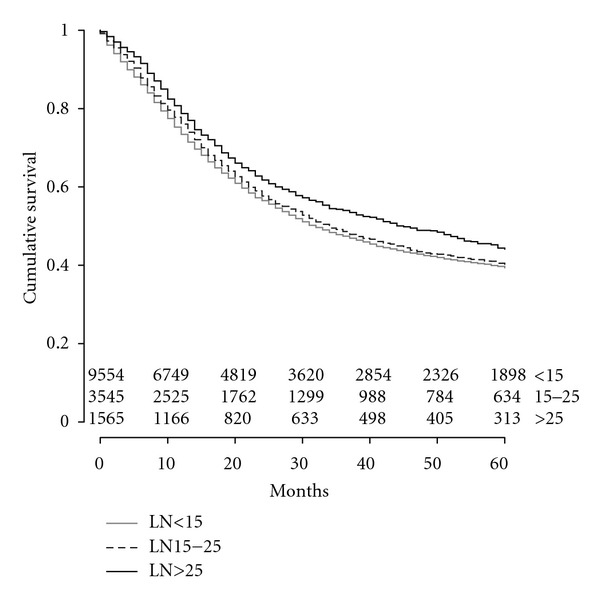
Kaplan Meier curve of disease specific survival by lymph node resection. Lymph node (LN).

An extended LND was also associated with improved OS by Kaplan Meier analysis. Median survival was 34 months for patients who had >25 LNs resected, 27 months if 15–25 LNs were resected, and 25 months if <15 LNs resected (*P* < 0.001). Five-year OS was 38% when >25 LNs resected, 33% with 15–25 LNs resected, and 31% if <15 LNs were resected (see Figure 1). Median DSS was also improved with a more extended LND: 45 months for patients with resection of >25 LN, 34 months for 15–25 LNs, and 32 months for <15 LNs (*P* < 0.001) (Figure 2). 

By Cox multivariate regression analysis, adjuvant XRT was independently associated with improved OS (HR 0.67, CI 0.64–0.71) and DSS (HR 0.69, CI 0.65–0.73) in patients with stages IB through IV (M0) undergoing resection for gastric cancer (see Tables 3 and 4). Notably, when extent of lymphadenectomy was included as a covariate in this analysis with patients stratified into groups according to the number of LNs resected (<15 LN, 15–25 LN, or >25 LN), the demonstrated OS and DSS benefit of adjuvant XRT persisted regardless of the extent of lymphadenectomy. In this model, a more extended LND (>25 LN) was also independently associated with improved OS and DSS when compared with a less extensive LND (<15 LN-OS HR 0.65, CI 0.60–0.69; DSS HR 0.62, CI 0.57–0.67 or 15–25 LN-OS HR 0.84, CI 0.78–0.91; DSS HR 0.81, CI 0.75–0.88).

Additional variables that were independently associated with improved OS and DSS included female gender (*P* < 0.001), race other than black (*P* < 0.001), younger age (≤60) (*P* < 0.001), and lower tumor stage (*P* < 0.001) as shown in Tables 3 and 4.

## 4. Discussion

The primary aim of our study was to determine whether adjuvant radiation therapy provides a survival benefit specifically to patients who have undergone an extended lymphadenectomy. To answer this question, we used data from SEER, a large, national database statistically powered to detect differences in OS and DSS. Using a multivariate analysis of over 15,000 patients, we demonstrate that an OS and DSS advantage persists in patients receiving adjuvant XRT even in the subgroup who have undergone an extended LND (>25 LN). The strength of this analysis lies in the inclusion of a large number of patients who underwent extended lymphadenectomy (*N* = 1,565). Although a subgroup analysis of patients who underwent D2 resection in INT-0116 was performed, the analysis was underpowered due to the small number of patients studied (*N* = 54).

The extent of lymphadenectomy accompanying gastrectomy remains controversial. An extended LND is thought to decrease locoregional recurrence and provide more accurate staging information. Although Japanese series have consistently shown a significant survival benefit with D2 LND, two large European randomized controlled trials, the Dutch Gastric Cancer Trial (DGCT) and the UK Medical Research Council (MRC) trial, failed to demonstrate this benefit [11–17]. In both of these trials, the morbidity and mortality of patients who underwent a D2 LND was significantly higher than patients who underwent a limited (D1) LND [17, 18].

However, a subset analysis of DGCT found a survival benefit of D2 LND for stages II and IIIA, a finding also demonstrated by a large, prospective German trial [16, 19]. Furthermore, a subset analysis of the MRC trial demonstrated superior survival for patients who underwent a D2 LND without splenectomy or pancreatectomy, indicating that the high-operative mortality may have masked the survival benefit of a D2 LND [17]. Retrospective reviews in the USA using SEER and the National Cancer Database have similarly demonstrated an improved OS after extended lymphadenectomy for patients with stages II–IV gastric cancer [20, 21]. 

The long-term results of DGCT have been recently released and indicate that D2 lymphadenectomy is associated with reduced locoregional recurrence rates and improved DSS, and the authors therefore recommend a modified spleen-preserving D2 lymphadenectomy [6]. In addition, the National Comprehensive Cancer Network (NCCN) has recently modified their recommendations to include a D2 lymphadenectomy for patients with resectable gastric cancer [22]. 

The D level of a lymphadenectomy is defined by the Japanese Research Society for the Study of Gastric Cancer (JRSGC) based on the location of the primary tumor in the stomach and the level of regional lymph node station involvement [9]. However, the number of lymph nodes removed can also be used to approximate the extent of LND. According to the Japanese literature, a radical lymphadenectomy corresponding to a D2 resection consists of removal of 26 or more nodes [23]. In a Korean study of 990 patients who underwent curative resection with negative margins and D2 lymphadenectomy, 87% of patients had >25 nodes resected [24]. In the Dutch randomized trial comparing D1 and D2 lymphadenectomy, the median number of nodes removed was 17 and 30, respectively [16]. This suggests that a D2 lymphadenectomy correlates with resection of approximately 25 or more LNs. Thus, when the anatomic nodal location is unknown, the level of LND (D1 or D2) can be approximated using the total number of resected LNs. It was necessary to use this definition in our analysis, as SEER data consists of the number of LN removed but does not include information about anatomic nodal stations.

In this study, we also found a survival benefit of adjuvant XRT after surgical resection in patients with stage IB through stage IV (M0) gastric cancer. Median survival improvement ranged from 8 months to 13 months depending on stage. These findings correspond to and confirm the results of the INT-0116 trial, which showed a 9 month median survival advantage with the use of adjuvant XRT and 5-FU [7]. 

Additionally, our study shows that a more extended LND in-and-of-itself results in improved OS and DSS with the survival benefit increasing as the extent of lymphadenectomy increases. These findings suggest that patients with gastric cancer should undergo a margin negative resection with an extended (>25 nodes or D2) lymphadenectomy and confirms that adjuvant radiation therapy is an important component of their treatment.

There are several limitations to this study. First, data on the use of chemotherapy is unavailable for analysis using the SEER database. Although adjuvant 5-FU chemotherapy and XRT was the standard of care during the study period, it is certainly possible that patients may have received an alternative adjuvant regimen. Some patients may have undergone surgery/5-FU only or surgery/XRT only. As these are not standard adjuvant therapy regimens, it is likely that the number of patients falling into these categories is low. In one study using SEER-Medicare data in which chemotherapy use is known, fewer than 15% of the 2,333 patients received surgery and either chemotherapy or radiation alone [8]. Further, the MAGIC trial, which demonstrated a benefit of perioperative chemotherapy was published in 2006, so it is unlikely that a significant proportion of patients received chemotherapy alone outside of a clinical trial during the dates of this study [25]. 

SEER data also does not include information about local or distant recurrence; therefore, disease-free survival (DFS) cannot be determined. Additionally, there is no information about margin status after resection or about the dose or details of the radiation administered. 

## 5. Conclusions

In summary, this study supports the use of adjuvant XRT in the treatment of gastric adenocarcinoma, as it appears to improve OS and DSS in patients with stage IB-IV (M0) gastric cancer. More importantly, our findings demonstrate that the survival benefit of XRT persists regardless of the extent of lymphadenectomy. This suggests that the benefit of XRT is not simply a mechanism to compensate for inadequate surgical clearance of disease, but is in itself critical for achieving locoregional control. Future prospective studies should include the use of adjuvant XRT and extended LND as independent variables to validate these findings.

## Figures and Tables

**Table 1:** Patient characteristics.

	No XRT		XRT		*P* value
	*N*	%	*N*	%
*N*	11852	100	3208	100	
Age					
≤60	2344	20	1286	40	<0.001
>60	9508	80	1922	60	
Sex					
Male	7452	63	2234	70	<0.001
Female	4400	37	974	30	
Race					
White	7926	67	2187	68	
Black	1364	12	368	11	0.284
Other	2562	22	653	20	
LN dissection					
<15 nodes	7731	67	1823	58	
15–25 nodes	2633	23	912	29	<0.001
>25 nodes	1141	10	424	13	
Grade					
I	820	7	86	3	
II	4213	36	919	29	<0.001
III	6583	56	2129	66	
IV	236	2	74	2	
Stage					
IA	2207	19	31	1	
IB	2717	23	503	16	
II	3146	27	1183	37	<0.001
IIIA	2041	17	817	25	
IIIB	395	3	212	7	
IV (M0)	1346	11	462	14	

**Table 2:** Overall survival by stage.

Stage	No XRT		XRT		*P* value
	*N*	Median survival (mo)	*N*	Median survival (mo)
All	11852	25	3208	28	<0.001
IA	2207	114	31	86	0.969
IB	2717	54	503	65	0.002
II	3146	21	1183	34	<0.001
IIIA	2041	14	817	23	<0.001
IIIB	395	11	212	19	<0.001
IV (M0)	1346	9	462	17	<0.001

**Table 3:** Cox multivariate regression analysis for overall survival.

Variable	HR	95% CI	*P* value
No XRT	1.00	Reference	<0.001
Adjuvant XRT	0.67	0.64–0.71	
Age			
≤60	1.00	Reference	<0.001
>60	1.49	1.42–1.57	
Gender			
Male	1.00	Reference	<0.001
Female	0.88	0.84–0.91	
Race			
White	1.00	Reference	
Black	1.06	0.99–1.13	0.075
Other	0.77	0.73–0.81	<0.001
Lymph nodes			
LN <15 : >25	0.65	0.60–0.69	<0.001
LN 15–26 : >25	0.84	0.78–0.91	<0.001
Stage			
IA	1.00	Reference	
IB	1.689	1.55–1.84	0.004
II	3.08	2.84–3.35	<0.001
IIIA	4.44	4.08–4.83	<0.001
IIIB	6.02	5.34–6.78	0.003
IV (M0)	7.14	6.52–7.82	<0.001

**Table 4:** Cox multivariate regression analysis for disease-specific survival.

Variable	HR	95% CI	*P* value
No XRT	1.00	Reference	<0.001
Adjuvant XRT	0.69	0.65–0.73	
Age			
≤60	1.00	Reference	<0.001
>60	1.26	1.19–1.33	
Gender			
Male	1.00	Reference	<0.001
Female	0.88	0.84–0.93	
Race			
White	1.00	Reference	
Black	1.04	0.97–1.11	0.307
Other	0.75	0.71–0.80	<0.001
Lymph nodes			
LN <15 : >25	0.62	0.57–0.67	<0.001
LN 15–26 : >25	0.81	0.75–0.88	<0.001
Stage			
IA	1.00	Reference	
IB	2.47	2.18–2.80	<0.001
II	5.38	4.78–6.05	<0.001
IIIA	8.32	7.38–9.39	<0.001
IIIB	11.31	9.74–13.12	<0.001
IV (M0)	13.78	12.16–15.61	<0.001
